# Barriers faced by people with disabilities in mainstream sports: a systematic review

**DOI:** 10.3389/fspor.2025.1520962

**Published:** 2025-02-03

**Authors:** Pablo Elipe-Lorenzo, Pelayo Diez-Fernández, Brais Ruibal-Lista, Sergio López-García

**Affiliations:** ^1^ Facultad de Educación, Universidad Pontificia de Salamanca, Salamanca, España; ^2^ Grupo de Investigación en Actividad Física, Deporte y Salud (GIADES), Facultad de Educación, Universidad Pontificia de Salamanca, Salamanca, Spain; ^3^ Unidad Consolidada de Investigación de Castilla y León, (UIC 382), Salamanca, Spain; ^4^ EUM Fray Luis de León, Universidad Católica de Ávila, Valladolid, España; ^5^ Facultad de Educación, Universidad de Salamanca, Salamanca, España

**Keywords:** disability, barriers, mainstream sports, inclusion, inclusive policies

## Abstract

**Introduction:**

Despite advances in inclusive policies and social awareness, the participation of people with disabilities (PwD) in mainstream sports remains limited due to numerous barriers. This systematic review seeks to identify and critically analyse the main obstacles hindering equitable participation of PwD in conventional sports, while proposing evidence-based strategies to overcome these challenges.

**Methods:**

Following PRISMA guidelines, a comprehensive search was conducted on Web of Science and SCOPUS databases, covering studies published between 2000 and 2024. After applying inclusion and exclusion criteria, 17 studies were selected for analysis.

**Results:**

The findings highlight major barriers, including insufficient training for coaches and sports club managers, negative and discriminatory attitudes, an entrenched ableist mindset, limited access to information, and a lack of accessible facilities. These factors collectively impede the active participation of PwD in sports.

**Discussion:**

To overcome these challenges, a coordinated approach is essential, encompassing attitude transformation, targeted training for sports personnel, the implementation of inclusive policies, economic incentives, and enhanced communication strategies. Additional recommendations include integrating universal design principles into sports facilities, establishing support networks and fostering a cultural shift in societal perceptions of disability.

**Systematic Review Registration:**

PROSPERO (CRD42024544589).

## 1 Introduction

The full and equitable inclusion of people with disabilities (PwD) remains a persistent challenge across various societal domains (1). Although progress has been made in raising social awareness about the importance of inclusion, significant challenges persist, hindering the quality of life and full development of PwD (2). This population faces multiple barriers, ranging from a lack of access to inclusive education to discrimination in the workplace. Additionally, the lack of specific regulations continues to perpetuate the exclusion of PwD, limiting their ability to fully contribute to society. Barriers faced by PwD encompass various domains, with the most prevalent being attitudinal, physical environments, transportation, policies, and inadequate support from personnel and service providers (3).

Disability is not a fixed or binary condition; rather, it is flexible and influenced by the individual's or family's strengths and limitations, as well as the supports available within their environment (4, 5). PwD have the same right to be included in the community and live independently, with the same choices as others (6). Therefore, it is essential to design interventions, services, and supports based on collaboration and a comprehensive understanding of disability that stems from both lived experience and specialized knowledge (4, 5).

Social inclusion through sports is a key strategy that ensures the active participation of all people, regardless of their abilities, in sporting activities within mainstream organizations (7). This inclusion significantly impacts quality of life and physical well-being (8), as well as psychological and emotional well-being (9), and contributes to the holistic development of the person (10). Recent studies highlight the unique ability of sports to transcend social barriers, providing inclusive opportunities that simultaneously foster skill development, build support network and independence among PwD (11, 12). This social aspect of sports significantly helps counteract the isolation often experienced by PwD, strengthening community cohesion and fostering a sense of belonging (13). Furthermore, it is vital to empower PwD with the autonomy to choose how, where, and with whom they want to engage in sports activities (14).

Despite some progress in inclusion, PwD still face significant barriers that restrict their access to sports in mainstream settings (6). There is currently no comprehensive synthesis that encompasses the barriers, limitations, and challenges faced by PwD in mainstream sports. This area remains underexplored in sports research, likely reflecting a lack of interest. This gap may stem from ableist perspectives that remain prevalent in academic discourse (15). Existing reviews on the barriers faced by PwD in sports environments include articles focused on contexts where regulatory adaptations are implemented and/or segregated settings are utilised (16–18). Nevertheless, promoting the participation of PwD in mainstream sports could be considered a strategy aligned with the principles of inclusive participation, as established in Article 30 of the Convention on the Rights of Persons with Disabilities (CRPD) (19).

The present study aims to fill this gap by conducting a systematic review that thoroughly examines these barriers. By identifying and analysing these obstacles, the study will facilitate the development of targeted tools and strategies to overcome them. These solutions will focus on promoting inclusion and fostering equitable participation, ensuring that segregation or regulatory adaptations are not the only alternatives. In doing so, we seek to promote a more inclusive and equitable sports environment, where PwD can participate freely, according to their preferences and without limitations.

## 2 Methodology

This study follows the methodological guidelines set by the Preferred Reporting Items for Systematic Reviews and Meta-Analysis (PRISMA) (20), recognized for their effectiveness in conducting systematic reviews. The methodology adopted, in line with PRISMA guidelines, is presented as a crucial component to ensure the integrity and transparency of the research.

This systematic approach provides a comprehensive framework for the search, selection, and synthesis of scientific literature, ensuring thoroughness and objectivity in reviewing the available evidence. Applying these methodological guidelines strengthens the validity and reliability of the results obtained in this study. The research was registered in the “International Prospective Register of Systematic Reviews” (PROSPERO) in 2024 (CRD42024544589).

### 2.1 Search strategy

A systematic search was conducted in the Web of Science (WOS) and SCOPUS databases, limiting results to works published from 2000 to the present. This restriction is based on the paradigm shift in human rights that began in the early 21st century, promoting the empowerment of people with disabilities as active members of an inclusive community (21).

The terminology related to “disability” is highly variable across research. To ensure comprehensive coverage, the most commonly used terms were included in the search strategy, such as disability, disabled people, people with disabilities, disabled, and functional diversity. Similarly, the concept of barriers was approached broadly, incorporating terms like limitations, obstacles, and challenges to encompass a wide range of difficulties reported in the literature. Therefore, the systematic search was conducted using Boolean operators (AND and OR) to structure a precise protocol, including the following terms: (“disability” OR “disabled people” OR “people with disabilities” OR “disabled” OR “functional diversity”) AND (“barriers” OR “limitations” OR “obstacles” OR “challenges”) AND (“sport”). This strategy was adopted to capture the diversity of expressions and perspectives present in the field, allowing for the identification of a broad range of results within the selected databases.

Specific exclusion criteria were applied during the selection of studies to ensure the coherence and relevance of the data collected. These criteria were rigorously applied to limit the study's scope to specific areas of interest and avoid including non-pertinent or potentially biased data, such as literature reviews, abstracts, editorial comments, and letters to the editors.

The criteria for inclusion or exclusion of articles were as follows:

Inclusion Criteria:

1. Articles written in English or Spanish.
2. Studies addressing perceived barriers to conventional sports environments experienced by people with and without disabilities, such as family members, coaches, therapists, etc., regardless of the sports context.
3. Research that explores the barriers faced by people with any type of disability, including intellectual, developmental, sensory, or physical disabilities.
4. Studies published in peer-reviewed journals.

Exclusion Criteria:

1. Sports aimed at high performance, pedagogical purposes, or segregated purposes (rehabilitation, home training, medical focus, therapies, elite sports, Paralympics, Special Olympics, Global Games).
2. Adapted sports. However, all support materials or individuals were considered integral to the person and therefore not classified as adapted sports. Any sport that modified the original rules was excluded.
3. Older adults whose disabilities were primarily acquired due to advanced age.
4. Systematic reviews, scoping reviews, mapping studies, meta-analyses or bibliometric analyses.

### 2.2 Procedure

Following the inclusion criteria previously outlined, individual reviews of each record were carried out by two reviewers. This approach provided greater consistency in the analysis and screening process. The selection of studies was independently conducted by the first and second authors of the article, following the predefined criteria. Any discrepancies that arose between the reviewers were resolved through consensus with the third and fourth authors.

**Figure 1:**
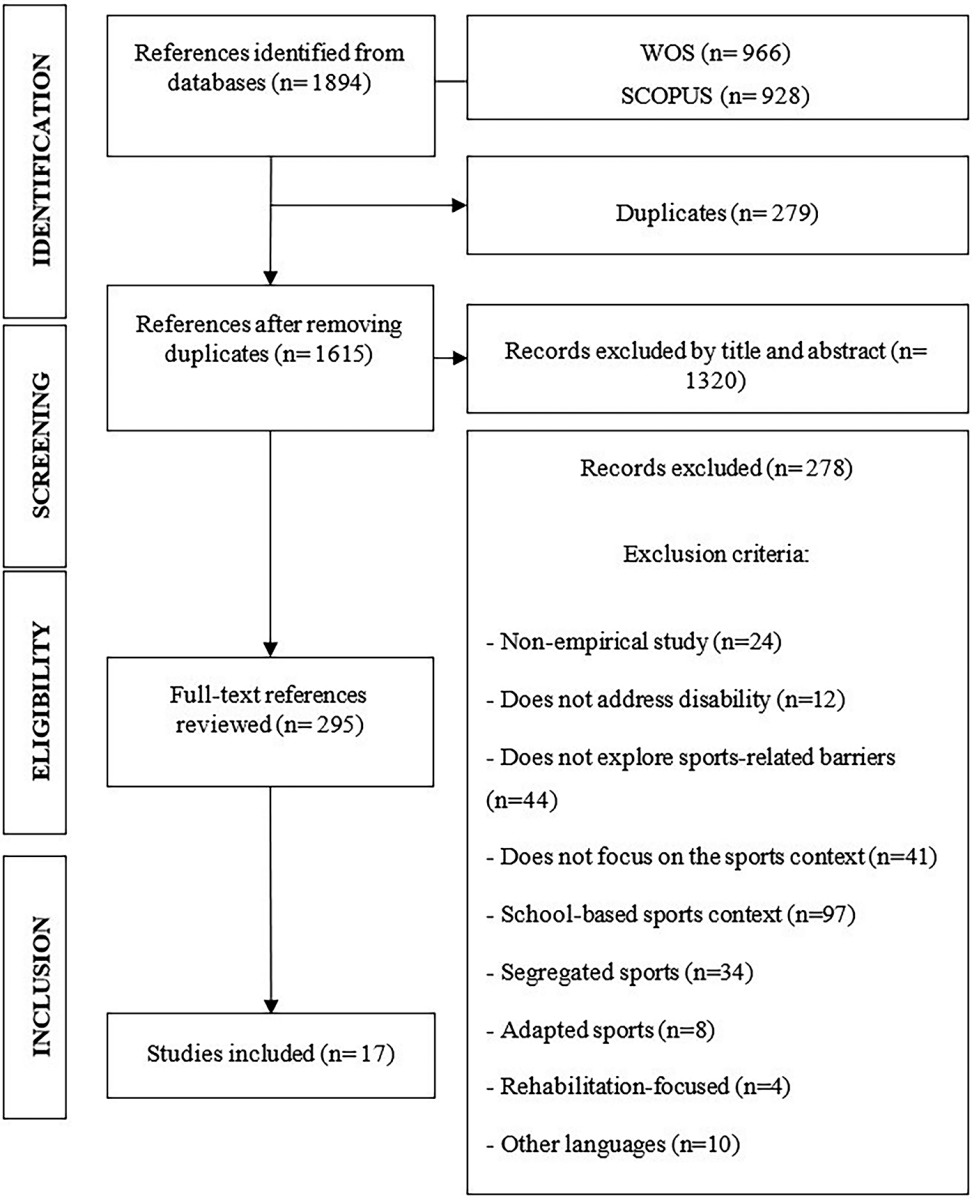
Flow diagram.

During the initial review, articles whose titles and abstract content did not relate to the topic were discarded. Subsequently, a more exhaustive reading of the literature was conducted to decide the inclusion and analysis of the final results. Figure 1 illustrates the four stages of the PRISMA Declaration: identification, screening, eligibility, and inclusion of the documentation (22).

The search process took place between March and May 2024. After the initial search and the registration of all identified files in WOS (*n* = 966) and SCOPUS (*n* = 928), a total of 1,894 documents were obtained. Duplicate articles were eliminated (*n* = 279) to avoid duplication problems, resulting in a total of 1,615 files.

The second phase of the protocol, as indicated by the PRISMA Declaration (22), is screening. In this phase, an exhaustive analysis of the documentation was conducted by reading the title and abstract of the articles selected in the first phase (identification). Out of 1,615 articles, after reviewing titles and abstracts, 1,320 were deemed irrelevant to the objectives of this study and were discarded. Subsequently, an in-depth review of the remaining 295 articles was carried out. After applying the eligibility criteria, those articles that did not meet the inclusion criteria were discarded (*n* = 278). Finally, the total number of documents included in this systematic review was 17 articles.

To analyse the information and data extracted from the 17 articles included in the review, an Excel sheet was created to compile details such as author and year, the number and type of participants, the protocol followed, and the results obtained. Additionally, the articles were categorized based on the characteristics of the barriers, lack of trained staff, lack of accessible programs, inaccurate understanding of inclusion, attitudinal barriers, parental barriers, ableist ideas, overly competitive approaches, emotional and psychological barriers, limited access to information, governmental and community organizations barriers, economic barriers and transport barriers, to address the specific objectives of this review.

## 3 Results

The sample used and analysed in various studies was highly diverse, encompassing a wide range of participants, including people with physical, intellectual, developmental, and sensory disabilities. Furthermore, the sample encompassed various stakeholders in sports programmes, such as coaches, family members, facility providers, planners and representatives from sports clubs and governing bodies responsible for sports provision and policy. Other participants included staff and volunteers from community organisations, therapists, clinicians, running guides and gym members without disabilities. These studies were conducted across several countries, including the Netherlands (23), Australia (24–27), New Zealand (28), the United Kingdom (29–33), Spain (34), Denmark (35), the United States (36–38), and Germany (39). They covered multiple sports disciplines, including rowing, cricket, boxing, tennis, and golf (30), as well as yoga, dance, judo, horseback riding, and open-water swimming (31). Other sports such as athletics (34, 36, 37, 39) and swimming or triathlon (29) were also included. Regarding the methods employed, the majority of studies (*n* = 15) adopted qualitative approaches, with semi-structured interviews serving as the principal data collection method. Of these, seven studies utilised individual interviews (24, 27, 29, 33, 34, 36, 37), while four relied solely on semi-structured focus groups (25, 31, 35, 39). Moreover, four studies used both individual interviews and focus groups (28, 30, 32, 38). Furthermore, one study implemented open-ended survey questions (27), and another incorporated observational participation alongside a case study design (30). In addition, mixed-methods approaches were identified in two studies, which integrated qualitative and quantitative methodologies; one combined online surveys with semi-structured individual interviews (26), while the other employed multiple-choice questionnaires, open-ended surveys, and structured individual interviews (23).

The systematic review identified various barriers faced by PwD when participating in mainstream sports. Table 1 presents the 17 documents selected for this analysis, showing the results obtained under the criteria established in the review process. Regarding the temporal distribution of the included studies, it was observed that only one article was published before 2010. Six articles were published between 2010 and 2020, while the remaining ten studies correspond to publications from 2020 onwards. This distribution suggests a growing interest and increased research output in recent years on the barriers PwD face in mainstream sports.

**Table 1:** Summary of selected studies.

Reference	Sample	Methodology/instruments	Results of barriers encountered
Adams et al. (23)	Quantitatively: Therapists (*n* = 243). Qualitatively: Therapists (*n* = 10), children with disabilities (*n* = 9) and their parents.	Mixed-methods approach (multiple-choice questionnaire and open-ended survey and structured individual interviews).	– LAP: Sessions not adjusted to the children's disabilities. – PB: Parents did not encourage their children to participate in sports. – LTS: Lack of opportunities in clubs and lack of knowledge by coaches.
Alcaraz Rodríguez et al. (34)	People with visual disabilities (*n* = 26) and guides (*n* = 23).	Qualitative study (semi-structured individual interviews).	– LAP and LAI: Lack of accessibility in the information provided by the organizers. – EB: Financial difficulty associated with registration fees. – LTS: Lack of specific training for organizing staff and support.
Ball et al. (36)	Runners with visual disabilities (*n* = 7) and guides (*n* = 4).	Qualitative study (semi-structured individual interviews).	– LAP: Difficulty finding suitable guides. – AB: Negative attitudes and lack of sensitivity from other runners.
Barr & Shields (24)	Parents of children with intellectual disabilities (*n* = 26).	Qualitative study (semi-structured individual interviews).	– FB: Family overprotection. – LAP: Lack of clubs that accept and incorporate al levels. – LTS: Insufficiently trained coaches. – AB: Preconceived notions, stereotypes, and the implications of using the term “disability."
Christiaens & Brittain (29)	Organizations responsible for sports provision and policy (*n* = 22) and individuals with physical, sensory, and intellectual disabilities (*n* = 9).	Qualitative study (semi-structured individual interviews).	– LAP and IUI: Lack of a strategic and proactive approach to inclusion by clubs. – LAP: Tendency towards segregation. – AB: Stereotypes and negative attitudes within clubs. – LTS: Lack of knowledge from coaches and club staff. – AI: Only those deemed capable of meeting non-disabled standards, typically individuals with mild disabilities, are considered for inclusion.
Dyer & Sandford (30)	Participants of MA, family members, coaches, club representatives, IMAS representatives, and government sports bodies (*n* = 142).	Qualitative study (active and observational participation in 85 sessions, three workshops, a case study, semi-structured individual interviews and one focus group).	– LAP and EPB: Lack of adequate social spaces due to the perception of difference and fear of participation. – IUI: Charitable approach to their participation. – OCA: Competitive and traditional image. – IUI: Negative language use.
Hall et al. (37)	Runners with visual disabilities (*n* = 5) and guides (*n* = 5).	Qualitative study (semi-structured individual interviews).	– AB: Exclusionary messages within running groups. – LAP: Inaccessible spaces. – EB: Financial difficulty. – AB: Negative attitudes and behaviours from non-disabled runners.
Hillan et al. (31)	People with visual disabilities (*n* = 7).	Qualitative study (semi-structured focus groups).	– TB: Lack of reliable and frequent public transportation. – EPB: Negative past experiences in sports that influenced their willingness to actively participate in the future. – LAI: Informational barriers.
Ives et al. (32)	People with physical, intellectual, developmental and sensory disabilities (*n* = 24).	Qualitative design (semi-structured individual interviews and focus groups).	– EB and TB: High cost of activities and transportation. – OCA: Preconceived images of sport as competitive and critical. – LAI: Lack of knowledge about available offerings. – LAI: Poor communication and ineffective modes of advertising. – GCOB: Lack of coordination between local government entities and community organizations. – EPB: Low self-esteem due to fear of social judgment.
Jones (38)	Parents of children with physical and developmental disabilities (*n* = 37).	Qualitative study (semi-structured individual and focus groups interviews).	– OCA: Highly competitive sports. – LAP and AB: Exclusion due to lack of behavioural and social skills. Lack of programmes aimed at fostering friendships. – LTS and AB: Negative attitudes of sport staff. – LTS: Lack of staff awareness about disabilities. – PB: Parents making decisions on behalf of PwD.
Kappelides et al. (25)	People with intellectual disabilities (*n* = 81) and staff and volunteers from community organizations (*n* = 10).	Qualitative study (semi-structured focus groups).	– LAI: Lack of access to information about sports programs due to cultural barriers. – LAI: Difficulty navigating computers. – AB and EPB: Discriminatory attitudes towards them had either caused discomfort or prevented their full participation. – EB and TB: Prohibitive costs and transportation limitations – LAP: Insufficient support. – GCOB and AB: Discriminatory attitudes within organizations. – AB and OCA: PwD are excluded from opportunities if it is assumed their participation might lead to the team's defeat.
Mulligan et al. (28)	People with physical disabilities (*n* = 21), facility providers (*n* = 17), planners (*n* = 15).	Qualitative study (semi-structured individual and focus groups interviews).	– EB and TB: High transportation and activity costs. – LTS: Qualified professionals. – LAP: Inaccessible facilities. – GCOB: Lack of inclusion policies.
Nikolajsen et al. (35)	Gym members without disabilities (*n* = 18).	Qualitative study (semi-structured focus groups).	– AB and LAP: Discrimination or restricted access. – AB and AI: Prejudices and ableist perceptions. – AB and IUI: Conflicting attitudes toward inclusion.
Pochstein (39)	Parents of children with intellectual disabilities (*n* = 25) and representatives of conventional clubs (*n* = 4).	Qualitative study (semi-structured focus groups).	– LAP and AB: Negative experiences when attempting to participate in sports clubs. – LAP: Lack of preparation and resources by clubs. – LTS and IUI: Unqualified coaches and lack of inclusion in programs.
Richardson et al. (33)	People with physical disabilities (*n* = 21).	Qualitative study (semi-structured individual interviews).	– LAP: Lack of accessibility in sports centres and inadequate equipment. – LAP and IUI: Cultural and aesthetic environment that is not inclusive. – AB and GCOB: Restrictive health standards and institutionalized ableism. – AB and EPB: Negative emotional and social experiences, including perceived judgment and lack of acceptance by others.
Shuttleworth et al. (26)	Quantitatively: Parents of children with physical, intellectual and developmental disabilities, and other syndromes (*n* = 58). Qualitatively: Same parents (*n* = 8).	Mixed-methods study design (online survey and semi-structured individual interviews).	– LAP: High staff turnover. – LTS: Coaches with low tolerance. – LTS and LAP: Sessions not tailored to individual needs. – LTS: Challenges related to the need of support. – AB: Poor attitudes from peers. – OCA: Pressure to move toward competitive gymnastics widened the gap between those with and without disabilities.
Wright et al. (27)	Clinicians (*n* = 6) and youth with disabilities (*n* = 28).	Qualitative study (semi-structured individual interviews and open-ended survey questions).	– EPB: Embarrassment, fear of failure, and fear of standing out negatively. – EPB: Low self-esteem and lack of motivation. – PB: Parental pressure. – LAP: Lack of inclusive and appropriate opportunities. – OCA and LAP: Difficulties with rules and the high level of competition. – AB and LTS: Poor attitude from peers and coaches. – TB and EB: Limitations with distance to sports centres, activity costs, and reliance on transportation.

LTS, lack of trained staff; LAP, lack of accessible programs; IUI, inaccurate understanding of inclusion; AB, attitudinal barriers; PB, parental barriers; AI, ableist ideas; OCA, overly competitive approaches; EPB, emotional and psychological barriers; LAI, limited access to information; GCOB, governmental and community organizations barriers; EB, economic barriers; TB, transport barriers.

One of the most significant barriers is the lack of trained staff and accessible programs, identified by several authors (23, 24, 28, 29, 31, 34, 35, 39). Additionally, a few several studies highlighted an inaccurate understanding of the concept “inclusion” (29, 30, 39).

Attitudinal barriers also play a crucial role, being cited as one of the main limitations by eleven authors in the sports environment (24–27, 29, 30, 33, 35–38). These include negative attitudes and prejudices, as well as a lack of genuine acceptance. Parental barriers, such as overprotection, were also identified (23, 24, 27, 38). These limitations are reinforced by ableist ideas and overly competitive approaches (23–25, 27, 29–31, 33, 35, 38, 39). Emotional and psychological barriers were also significant, driven by discriminatory attitudes and negative stereotypes (25, 27, 29, 32, 37, 39). Furthermore, ten studies mentioned physical barriers and access to information, such as a lack of accessible facilities and a shortage of inclusive opportunities (24–28, 31–35, 37).

On the other hand, barriers imposed by governmental and community organizations were identified, including the absence of effective inclusive policies and insufficient coordination (28, 29, 32, 39). Finally, economic barriers related to the costs of participation and inadequate transportation also emerged as significant obstacles (25, 27, 31, 32, 37).

## 4 Discussion

This review sought to identify the barriers that people with disabilities (PwD) face when participating in mainstream sports. To meet this objective, 17 studies were analysed to explore the difficulties these individuals encounter. Understanding these obstacles is essential for reducing existing barriers and fostering inclusive communities. As Lewis and Richardson (40) suggest, communities are spaces for transformation. Therefore, it is crucial to implement the necessary adjustments to ensure they are accessible and inclusive, enabling all individuals to lead full, meaningful lives. In this context, sports clubs must critically reflect on the challenges they present in order to achieve true inclusion (30).

The results of this study reveal a series of barriers that reflect an intersection of challenges requiring a comprehensive approach. Sports clubs often lack the experience and preparation necessary to effectively integrate PwD, a challenge that manifests in various ways. The study found confusion in sports clubs regarding the meaning of inclusion and disability (29, 30, 39). In this sense, families of PwD often perceive that their children are not welcome in mainstream clubs, reinforcing exclusion (39). Moreover, the available sports offerings often fail to consider individual preferences, mistakenly assuming that all participants are interested in the same activities, which are typically offered in segregated environments (31). Pearce & Sanderson (41) argue that although sports activities for PwD are often labeled as “inclusive,” they are not truly so. Offering separate or segregated opportunities, even if they are equitable, does not constitute true inclusion. Furthermore, achieving inclusion in mainstream environments requires active and genuine participation (12).

Moreover, findings indicate that sports facilities often lack appropriate infrastructure and equipment, significantly limiting the participation of PwD (28, 33). Discrimination or restricted access prevents PwD from enjoying the same opportunities as other individuals (24, 27, 31, 35). This exclusion is exacerbated by the surrounding cultural and aesthetic environment (33). To address these barriers, Lid (42) proposes implementing strategies based on universal design, which would ensure more equitable access and improve PwD participation in both sports and social activities. This approach must be interdisciplinary, considering both physical and relational barriers. Similarly, Yi et al. (43) recommend implementing universal design indicators to assess the management of sports facilities, ensuring greater accessibility and improved service quality. Additionally, the lack of public transportation, dependence on others for rides, and the costs associated with participating in sports activities further limit sports inclusion (25, 27, 31, 32, 37). Misener & Darcy (14) suggest that these barriers can be mitigated by establishing strong support networks within sports clubs. Organized systems, such as ride-sharing or volunteer-driven transportation solutions, can effectively address transportation challenges and foster greater participation.

In addition, several studies highlighted the insufficient training and lack of support from coaches, who often lack the skills and knowledge needed to appropriately adjust sports practice (23, 24, 26, 28, 29, 34, 35, 39). This lack of preparation to lead inclusive groups results in negative attitudes or fears towards inclusion (39). Therefore, it is essential for mainstream sports clubs to receive specific training to create supportive environments (44, 45). This support should include understanding and meeting individual needs, fostering goals, ensuring the availability and access to support, knowledge of the support systems, the presence of competent providers, maintaining consistency and stability in supports, and ensuring proper coordination and management (11, 46).

One of the key challenges identified in this study is the presence of attitudinal barriers and a lack of understanding within the sports environment. These barriers include intolerance and insensitivity towards certain behaviours, along with a limited awareness of the specific needs of PwD (26–28, 33, 36). Furthermore, stereotypes and negative attitudes persist, exacerbating these difficulties and significantly affecting inclusion (24, 29). In addition, parental attitudes are often overprotective and exercise constant supervision. In some cases, they fail to adequately encourage their children to participate in sports activities (20) or impose their own preferences without considering their children's desires (27, 38). These dynamics can reduce self-determination, affect self-esteem, and lead to poor performance throughout life (47, 48).

On the other hand, perceptions in the sports environment often align with a charitable view of disability, where non-disabled players assume the role of “volunteers” reinforcing unequal power dynamics (30). For inclusion to be effective, it is crucial that players with and without disabilities are treated as equals, avoiding any patronizing behaviour (19, 44). This approach not only promotes more equitable interaction but also fosters the autonomy and empowerment of all players. In this sense, ableism influences how PwD are perceived and treated in sports settings, often emphasizing charity or paternalism (49). Our results show that an ability-centric approach, lack of acceptance, and prejudice are factors that limit the participation of PwD in sports (25, 33, 35). Sport clubs frequently implement ableist practices that do not represent true inclusion, as they require PwD to meet normative standards (23, 27, 29, 31, 38, 39). As children with disabilities grow, the gap in physical and cognitive abilities compared to their peers widens (24). The study by Dyer and Sandford (30) reveals that some non-disabled people prefer only to “support” players with disabilities without actively participating in sports themselves, as they do not view the sport as “challenging”. This perspective is related to the belief that participants with disabilities would have a “lower” skill level. According to Brittain et al. (49), such ableist perceptions reinforce the idea that PwD cannot fully participate in challenging sports, once again limiting their self-determination in sports environments. Similarly, several studies found that one of the emotional barriers preventing participation was the fear of failure, along with the fear of being socially judged and not being recognized as full individuals (25, 27, 31, 32, 37, 39). Thus, a cultural shift is essential—one that embraces diversity and views individuals based on the qualities, rather than stereotypes, recognizing their full potential for participation. Media, educational institutions, and sports organizations must promote a more positive and authentic representation of PwD (45).

Politically, the creation of a truly inclusive sports environment faces significant obstacles due to the lack of coordination between governmental entities and community organizations, as well as the absence of mandatory inclusive policies with adequate funding (28, 32). The implementation of inclusive policies is often affected by ableist interpretations of disability by those responsible for enforcing them (29). Misener & Darcy (14) argue that the barriers faced by PwD stem from organizational structures and management practices, rather than a lack of interest on their part. There is also a considerable gap between sports associations for PwD and the mainstream sports system, which limits inclusion opportunities and reinforces the exclusion of these groups (39). The management of community sports must be a shared responsibility between organizations specialized in PwD and mainstream sports organizations (50). Therefore, local governments must implement sports policies that support these organizations within their communities (51). Moreover, national councils and bodies must go beyond accessibility regulations, adopting a comprehensive approach that operates at all levels of society. This approach should focus on inclusive strategies that eliminate discriminatory behaviours and promotes full participation (29, 45). In addition, it is essential that these measures developed in close collaboration with PwD, to ensure they are tailored effectively to meet their needs (45).

Finally, access to information about inclusive sports opportunities represents a significant barrier. The lack of clear and accessible information, both in physical and digital formats, makes it difficult for PwD to become aware of the opportunities available (31). The studies by Ives et al. (32) and Kappelides et al. (25) highlight that difficulties accessing information online and the lack of advertising in accessible formats leave many PwD dependent on third-party knowledge. This hinders self-determination processes (46). Similarly, Tsai and Fung (52) argue that organizations fail to manage and provide the necessary information on how and where to participate. In this sense, our findings show that ineffective communication and inadequate advertising contribute to limited awareness of the available sports offerings (32, 34, 37). This information deficit leads to feelings of exclusion and reinforces the social barriers faced by PwD (31). Comella et al. (53) suggest that sports organizations should establish stronger collaborations between national, state, and local levels to provide more accessible information.

## 5 Limitations

Among the limitations identified in this study is the predominance of research with a qualitative focus, which, while providing a deep understanding of individual experiences, may limit the generalizability of the findings to broader populations. Only two studies utilized mixed methods, which restricts the integration of qualitative and quantitative data.

Another significant limitation is the geographical diversity of the included studies, with a substantial concentration in countries like the United Kingdom and Australia, and limited representation from other regions, especially low-income countries.

These limitations underscore the need for future studies to adopt mixed methods approaches and consider a more diverse geographical representation to offer a more comprehensive and generalizable perspective on the barriers faced by PwD in mainstream sports.

## 6 Conclusions

This systematic review reveals the complex and multifaceted barriers faced by PwD in mainstream sports, showing that these challenges go well beyond mere physical access. The findings emphasize the significant impact of societal attitudes, structural inequalities, and policy gaps that contribute to ongoing exclusion. To foster true inclusion, sports organizations must adopt comprehensive strategies addressing diversity, equity and accessibility. Education and training for sports staff, coaches, and administrators are crucial in creating environments that enable full participation of PwD.

Equally important are effective communication strategies to ensure PwD have access to clear and accessible information about inclusive sports opportunities, by empowering them to make informed decisions about their involvement. At the policy level, governments must enforce mandatory inclusive policies and provide adequate funding to support initiatives aimed at increasing accessibility and inclusion in mainstream sports. Furthermore, collaboration between governmental bodies, mainstream sport clubs and associations for PwD is essential to ensure consistent implementation of these policies across national and local levels. By tackling these interconnected barriers through coordinated efforts across all levels, sport can open the door to a more inclusive and equitable society, empowering PwD to reach their full potential as integral and active members of the community. Finally, future studies should focus on developing tools and strategies that make mainstream sports environments truly inclusive for PwD. This involves identifying effective interventions to address the barriers highlighted in this study, while ensuring accessibility and equity.

## Data Availability

The original contributions presented in the study are included in the article/Supplementary Material, further inquiries can be directed to the corresponding author.
